# KPP: KEGG Pathway Painter

**DOI:** 10.1186/1752-0509-9-S2-S3

**Published:** 2015-04-15

**Authors:** Ganiraju Manyam, Aybike Birerdinc, Ancha Baranova

**Affiliations:** 1 School of Systems Biology, George Mason University, Fairfax, VA - 22030, USA; 2 The UT MD Anderson Cancer Center, Houston, TX - 77030, USA; 3 Research Centre for Medical Genetics RAMS, Moscow, Russia

**Keywords:** Data analysis, Data mining, Gene expression, Gene regulation, Visualization, Knowledge-based algorithms

## Abstract

**Background:**

High-throughput technologies became common tools to decipher genome-wide changes of gene expression (GE) patterns. Functional analysis of GE patterns is a daunting task as it requires often recourse to the public repositories of biological knowledge. On the other hand, in many cases researcher's inquiry can be served by a comprehensive glimpse. The KEGG PATHWAY database is a compilation of manually verified maps of biological interactions represented by the complete set of pathways related to signal transduction and other cellular processes. Rapid mapping of the differentially expressed genes to the KEGG pathways may provide an idea about the functional relevance of the gene lists corresponding to the high-throughput expression data.

**Results:**

Here we present a web based graphic tool KEGG Pathway Painter (KPP). KPP paints pathways from the KEGG database using large sets of the candidate genes accompanied by "overexpressed" or "underexpressed" marks, for example, those generated by microarrays or miRNA profilings.

**Conclusion:**

KPP provides fast and comprehensive visualization of the global GE changes by consolidating a list of the color-coded candidate genes into the KEGG pathways. KPP is freely available and can be accessed at http://web.cos.gmu.edu/~gmanyam/kegg/

## Background

High-throughput technologies became common tools to decipher genome-wide changes of gene expression (GE) patterns or relative protein abundance. Typical output of these large-scale studies is represented by the list comprised of hundreds of gene candidates with attached quantitative labels. Functional analysis of these gene lists is a daunting task as it requires regular recourse to the public repositories of biological knowledge or use of expensive databases of manually curated biological annotation [1,2]. On the other hand, in many cases researcher's inquiry can be successfully served by a comprehensive glimpse.

Functional analysis of markers identified from large-scale datasets can be performed using a wide variety of bioinformatics tools. As microarrays became a common tool to decipher global gene expression, centralized systems like Gene Expression Omnibus (GEO), ArrayExpress was developed to congregate the valuable profile data [3,4]. An analysis of combined datasets generated in independent microarray experiments (so-called "microarray meta-analysis"), is often being employed [5], for example, to develop biomarker panels or to extract insights into the pathogenesis of various chronic diseases [6] including human malignancies [7]. Meta-analysis lead to an increase of the complexity in microarray analysis; therefore, sophistication of subsequent functional analysis also increased. Gene Ontology (GO) and other pathway-centered types of analysis became indispensable [8].

KEGG (Kyoto Encyclopedia of Genes and Genomes) is a compendium of databases covering both annotated genomes and protein interaction networks for all sequenced organisms. Its integral part, KEGG PATHWAY, is a compilation of manually verified pathway maps displaying both the molecular interactions and the biochemical reactions [9]. The recent version of this database includes a complete set of pathways related to signal transduction and other cellular processes [10]. The extensive collection of the pathways at KEGG can be utilized for the rapid graphical evaluation of the functional relevance of the observed changes in GE patterns. This will save the precious time of the expert biologists and bioinformatics specialists.

Pathways assembled into the KEGG database are displayed as semi-static objects that can be manipulated using tools like KGML and KEGG application programmable interface (API) [11,12]. KEGG API provides a routine that highlights specified genes within the particular metabolic pathway (http://www.genome.jp/kegg/tool/color_pathway.html). Similar task may be also executed using G-language Genome Analysis Environment [13]. Both approaches work on the pathway by pathway basis. Another tool, Pathway Express, calculates the pathway-wise impact of differentially expressed genes based on normalized fold change and depicts the pathways with differentially expressed genes [14]. However, the fold-change approach and its associated standard t-test statistics usually produce severely over-fitted models. A number of recently developed approaches generate gene rankings dissociated from the fold change estimates [15,16]. An analysis of these gene lists may benefit from the binary graphical mapping of upregulated and downregulated elements within the complete collection of pathway maps. Resulting graphical pictures may be helpful both as tool for a quick assessment of the functional relevance of a gene list and as a set of the snapshots easily convertible into the illustrative material for presentations or manuscript figures.

With this notion, here we present a web-based tool, KEGG Pathway Painter (KPP). KPP performs a batch painting of relevant pathways according to the uploaded lists of up-regulated and down-regulated genes in KEGG. KPP returns a set of images that give a holistic perspective to the functional importance of the change in the GE patterns revealed by a given high-throughput experiment and facilitate the extraction of the biological insights.

## Implementation

KPP was implemented using PERL/CGI. Pathways assembled into the KEGG database are displayed as semi-static objects that can be manipulated using tools like KGML (KEGG Markup Language) and KEGG API (Application Programming Interface). The API allows access to the resources stored in KEGG system in an interactive and user-friendly way (http://www.genome.jp/kegg/rest/).

KEGG Pathway Painter (KPP) accepts the up-regulated and down-regulated gene lists as two different text files containing the gene identifiers of any sequenced organism. Permitted identifiers include GenBank id, NCBI GENE id, NCBI GI accession, Unigene ID and Uniprot ID. Conversion of the gene identifiers, extraction of the corresponding pathway and their painting is performed by specific API routines. The KPP processes data through direct interface to the KEGG database, and therefore, the KPP painted pathways are always up-to date with reference to KEGG knowledgebase. In KPP, genes of interest can be also highlighted with user-specified foreground and background colors allowing easy visual differentiating of up- and down-regulated genes.

Mapped genes are automatically consolidated within each pathway. The number of the KPP returned pathways could be filtered by either the total number of the painted genes in a given pathway or the ratio of painted genes to the total number of genes in a given pathway. The chosen pathways passing the criteria on filter are color coded according to users' preferences. Users can browse through these high-resolution pathway images along with gene information and an archive of the painted pathways can also be saved for future reference. After completion of the query, the URL to the index of resulting output images is e-mailed to the user along with the job summary.

## Results and Discussion

The motivation for the development of KPP came up from the idea to build a user-friendly, platform-independent and simple tool to visualize the placement of genes in their associated pathways. The simplicity of KPP is due to the acceptance of gene identifiers without reference to respective microarray platform. This isolation enhances its utility for the studies of the data from RealTime-PCR or medium-throughput platforms or even for validation of the various hypotheses concerning an involvement of the groups of genes in one or another biological process.

**Figure 1:**
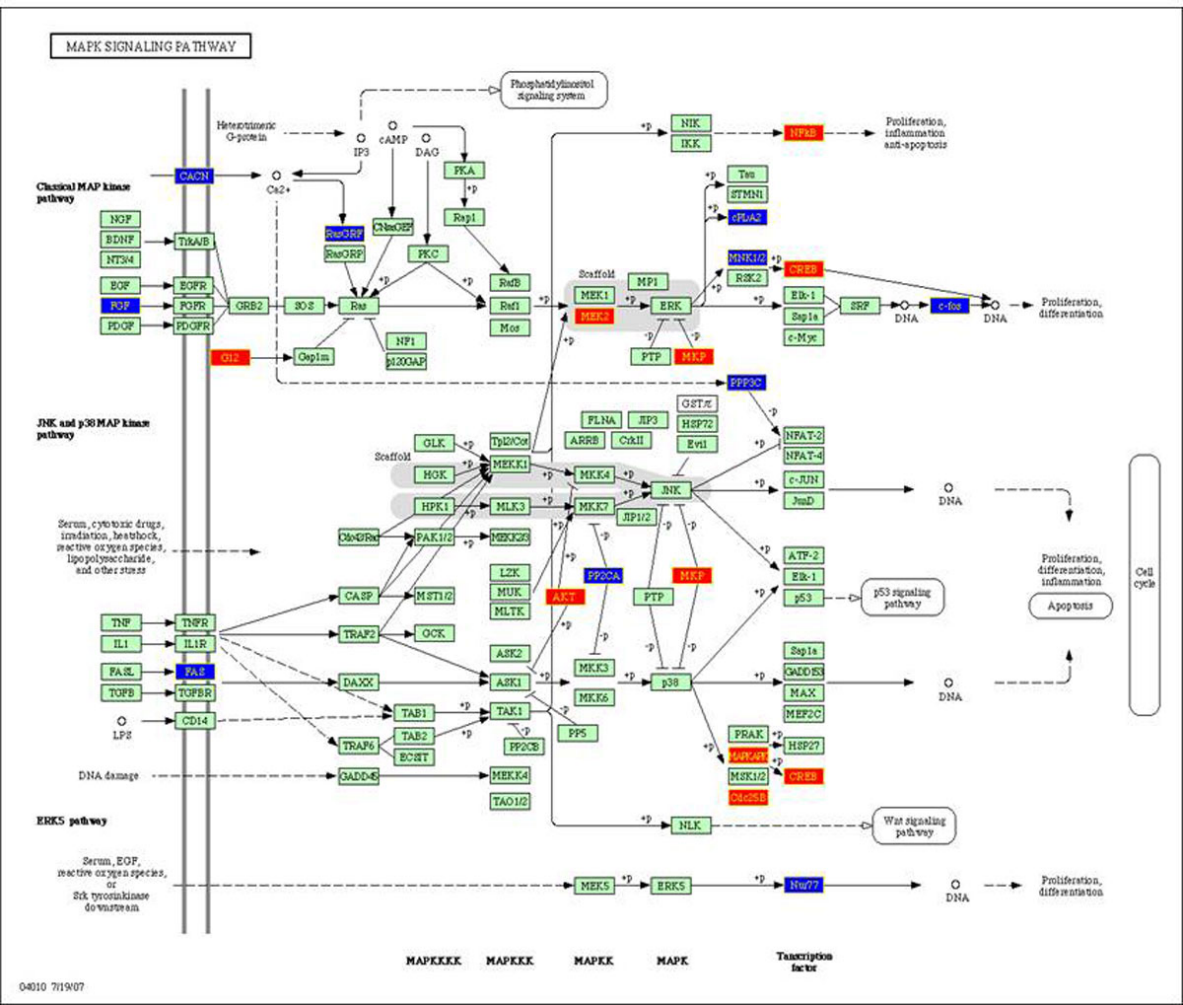
**Image of the MAPK signaling pathway painted by KPP according to the imported list of genes differentially expressed in the prostatic carcinoma as compared to normal prostate**. Red and blue boxes represent up- and down- regulated genes, respectively. The genes in green background represent the species specific genes (*Homo sapiens*, in this case).

This utility of KPP was demonstrated by highlighting of cell cycle related events using the publicly available prostate carcinoma dataset (GDS1439) [17] from the NCBI GEO database (see Figure 1), by aiding the selection of the mutations and epigenetic events to be tested as a companion diagnostics of treatment susceptibility and resistance in non-small lung carcinoma patients (not shown) and an analysis of the host-associated risk factors associated with lack of sustained virological response (SVR) in various cohorts of HCV patients [18,19].

In one of these examples, KPP-aided visual parsing the pathways encompassing molecular components relevant to HCV pathogenesis allowed to pinpoint the Janus kinase-signal transducers and activators of transcription signaling cascade as the major pathogenetic component responsible for not achieving SVR [18], a conclusion that was later confirmed in *in vitro *experiments with blocking antibodies, a pharmacological inhibitor, and siRNAs [20].

In another example, KPP allowed to visualize a sustained pattern of treatment-induced gene expression in patients carrying interferon/ribavirin-responding IL28B genotype C/C, while in patients with therapy-resistant IL28B T* genotype, the background pre-activation of interferon-dependent genes precluded further therapeutic boost [19]. Thus, KPP provided a critical insight into the lower rate of SVR observed in these patients. Furthermore, KPP analysis revealed LI28B genotype independent role of SOCS1 in therapeutic response [19]. This KPP-aided hypothesis was later investigated both in vitro experiments showing that SOCS1 acts as a suppressor of type I IFN function against HCV [21] and in serum samples interferon/ribavirin-treated Hepatitis C patients who demonstrated that methylation-based silencing of SOCS-1 is associated with better therapeutic response [22]. Thus, KPP was indispensable in acquiring mechanistic insights into the differential therapeutic response in Hepatitis C infected patients.

The major fetching point of the KPP tool lies in its tight connection with the KEGG database, as this will allow for the pathway visualization of every sequenced organism. However this flexibility comes at the cost of possible KEGG-attributed delay of the data transfer, the resultant tool is substantially more convenient for the user than the tools embed into existing pathway analysis environment, for example, Cytoskape (http://www.cytoscape.org/). Another commonly used pathway parsing tool, Reactome Skypainter (http://www.reactome.org/), is restricted to underlying knowledge base and, therefore, limits the potential set of insights to be extracted.

It is important to note that the painting of individual pathways can be performed through by the KEGG website itself (http://www.genome.jp/kegg/), however, the practicality of KPP is in its comprehensive visual representation of up- and downregulated genes in the KEGG dataset as a whole. In other words, KPP allows one to extract immediate and visual insights about cumulative change in each pathway under scrutiny. Users can browse through high-resolution pathway images and download an archive of the painted pathways that may be used as figures for upcoming manuscripts.

## Conclusion

In summary, KPP provides fast and comprehensive visualization of the global GE changes by consolidating a list of the color-coded candidate genes into the KEGG pathways.

## List of abbreviations

KPP - KEGG Pathway Painter

GE - Gene Expression

KGML - KEGG Markup Language

API - Application Programming Interface

SVR - Sustainer Virological Response

## Competing interests

The authors declare that they have no competing interests.

## Authors' contributions

All three authors contributed to the study design, interpretation of results and producing the manuscript. All authors read and approved the final manuscript.

## Declarations

Open access fees were covered by funds of College of Science, George Mason University.

This article has been published as part of *BMC Systems Biology *Volume 9 Supplement 2, 2015: Selected articles from the IX International Conference on the Bioinformatics of Genome Regulation and Structure\Systems Biology (BGRS\SB-2014): Systems Biology. The full contents of the supplement are available online at http://www.biomedcentral.com/bmcsystbiol/supplements/9/S2.
